# Landslides and dam damage resulting from the Jiuzhaigou earthquake (8 August 2017), Sichuan, China

**DOI:** 10.1098/rsos.171418

**Published:** 2018-03-28

**Authors:** Bo Zhao, Yun-sheng Wang, Yong-hong Luo, Jia Li, Xin Zhang, Tong Shen

**Affiliations:** State Key Laboratory of Geohazard Prevention and Geoenvironment Protection, Chengdu University of Technology, Chengdu, Sichuan 610059, People's Republic of China

**Keywords:** Jiuzhaigou Ms 7.0 earthquake, landslides, dam damaged, failure mode, distribution law

## Abstract

At 21.19 on 8 August 2017, an Ms 7.0 earthquake struck the Jiuzhaigou scenic spot in northwestern Sichuan Province, China. The Jiuzhaigou earthquake is a strike-slip earthquake with a focal depth of 20 km at 33.20° N and 103.82° E, and was caused by two concealed faults. According to emergency investigations and remote sensing interpretations, the Jiuzhaigou earthquake triggered 1780 landslides, damaged one dam (Nuorilang Waterfall) and broke one dam (Huohua Lake). The landslides mainly occurred in the Rize Valley and Shuzheng Valley and in Jiuzhai Paradise. The landslides involved hanging wall and back-slope effects, and the slope angle, slope aspect, seismic faults and valley trend were obviously related to the occurrence of the landslides. Specifically, most of the landslides were shallow landslides, rockfalls and rock avalanches and were small in scale. The failure modes of landslides mainly include wedge rock mass failure, residual deposit failure, relaxed rock mass failure and weathered rock mass failure. The initial low stability of the dam coupled with the topographic effect, back-slope effect and excess pore water pressure led to damage to the Nuorilang Waterfall dam.

## 1. Introduction

The Jiuzhaigou earthquake occurred at 21.19 local time (13.19 UTC) on 8 August 2017, at the Jiuzhaigou scenic spot in Sichuan Province, China. This earthquake had a magnitude of Ms 7.0 and a focal depth of 20 km at 33.20° N and 103.82° E [1]. The epicentre of the Jiuzhaigou earthquake is located in the eastern segment of the Tazang fracture zone and approximately 293 km from Chengdu City. By 20.00 on 13 August 2017, the Jiuzhaigou earthquake had resulted in 25 fatalities, 525 injured persons, 76 671 damaged rooms and a total estimated economic loss of more than 140 million RMB [2]. The earthquake triggered various types of geohazards, including landslides, dam break and dam damage.

Earthquake-triggered geohazards have aroused widespread concern worldwide as they can result in significant casualties and economic losses [3–7]. The damage caused by earthquake-triggered landslides may be more severe than the damage caused by the earthquake itself, particularly in mountainous areas, because they can bury villages, block roads and rivers, pose a serious threat to disaster relief and delay resettlement and reconstruction of the disaster area [8]. Reconstructing and reducing the damage caused by future earthquakes is very important for clarifying the distribution law of geohazards and their failure modes as soon as possible after an earthquake occurs.

On the second day of the Jiuzhaigou earthquake, our group entered the earthquake area and carried out an emergency investigation of geohazards. In addition, an aftershock monitoring scheme was also carried out to study the dynamic response in Jiuzhaigou region. Our study focuses on the characterization of the distribution and nature of landslides induced by the Jiuzhaigou earthquake and makes a comparison of these data with previous observations made during the 2008 Wenchuan Ms 8.0 earthquake and 2013 Lushan Ms 7.0 earthquake. This paper presents the preliminary results of an inventory map of the landslides triggered by the Jiuzhaigou earthquake in Sichuan Province, China. This study also aims to contribute to the further understanding of the characteristics of landslides and dam damage in the Jiuzhaigou region. In addition, dynamic response, obtained from aftershock monitoring, is used to discuss the failure cause of geohazards (landslide and dam damage) coupled with their characteristics.

## 2. Geological background

### 2.1. Regional geological setting of the Jiuzhaigou earthquake

**Figure 1.**
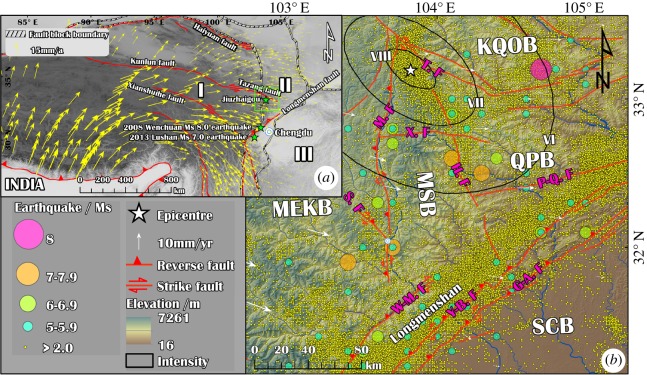
Simplified regional tectonic map of the Jiuzhaigou earthquake. (*a*) Map showing the topography of the Tibetan Plateau and the location of (*b*). (*b*) A simplified structural map with shaded topographic relief and showing the earthquake distribution. I: Tibetan fault block region; II: East boundary zone of I: III: Yangtze zone; MEKB: Maerkang Block; SCB: Sichuan Basin; MSB: Minshan Block; QPB: Qingchuan–Pingwu Block; KQOB: Kunlun–Qinling Orogenic Belt; G-A.F: Guanxian-Anxian fracture; Y-B.F: Yingxiu-Beichuan fracture; W-M.F: Wenchuan-Maoxian fracture; P-Q.F: Pingwu-Qingchuan fracture; H.F: Huya fracture; T.F: Tazang fracture; X.F: Xueshan fracture; M.F: Minjiang Valley fracture; S.F: Singpinggou fracture. The GPS velocities were downloaded from Zhao *et al.* [9].

The lateral extrusion of the Tibetan Plateau is widely accepted to be resisted by the rigid Sichuan basin to the east, resulting in the uplift of the Longmenshan region and the Minshan Block (figure 1*a*). The Minshan Block is located in the northwest triangle fault block of Sichuan Province (figure 1*b*). The average velocity (2009–2014) of the Minshan Block is approximately 6–10 mm yr^−1^ and is depicted by a primarily northeastward extrusion.

The Minshan Block is a key tectonic element of the earthquake occurrence. The Minshan Block is bordered by the Longmenshan Range fractures in the south, the Huya fracture in the east, the Tazang fracture in the north and the Minjiang fracture in the west. The rigidity of the block is relatively greater than that of the blocks in adjacent regions. The northeastward movement of the block pushed by regional maximum geo-stress is limited when it suddenly tapers off near the east triangle end with a bottle-neck effect, which results in an area with concentrated geo-stress that leads to many earthquakes (figure 1*b*) [10].

As shown in figure 1*b*, there is an obvious seismic belt along the boundaries of the Minjiang block; many great earthquakes (Ms ≥ 7.0) occurred along the boundaries of Minjiang block, such as the 1933 Diexi Ms 7.5 earthquake (east boundary), 1976 Songpan Ms 7.2 earthquake (west boundary) and 2008 Wenchuan 8.0 earthquake (south boundary).

The Jiuzhaigou earthquake is located at the north edge of the Minshan Block (figure 1*b*). Although several earthquakes were distributed along the northern edge of the Minshan Block, no large earthquakes occurred. At 21.19 local time (13.19 UTC) on 8 August 2017, an earthquake with a magnitude of Ms 7.0 and a focal depth of 20 km occurred at the north edge of the Minshan Block (figure 1*b*).

### 2.2. Seismicity characteristics

**Figure 2.**
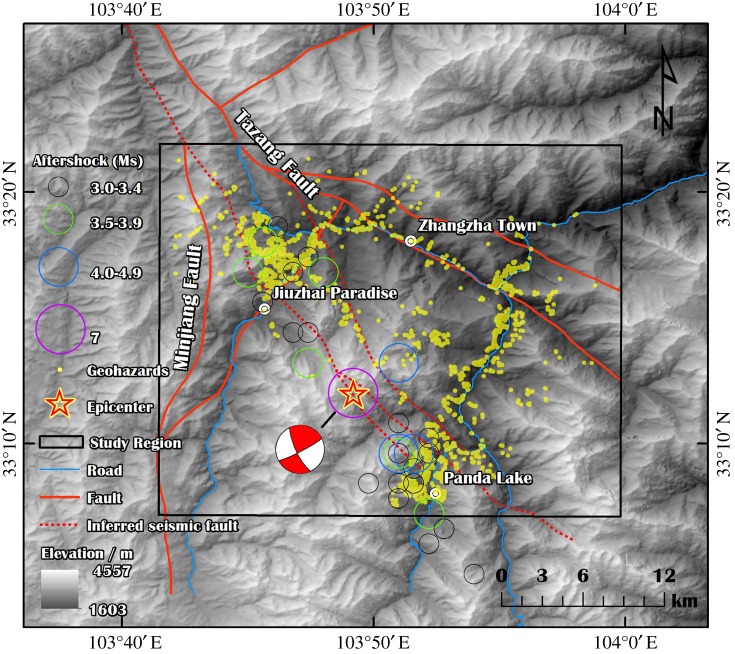
The distribution of aftershocks and the focal mechanism of the Jiuzhaigou earthquake. The seismic fault is provided by Prof. Li Yusheng [11].

According to the focal mechanism solution of the Jiuzhaigou earthquake, the Jiuzhaigou earthquake is a strike-slip earthquake with a focal depth of 20 km at 33.20° N and 103.82° E, as shown in figure 2 [12].

**Figure 3.**
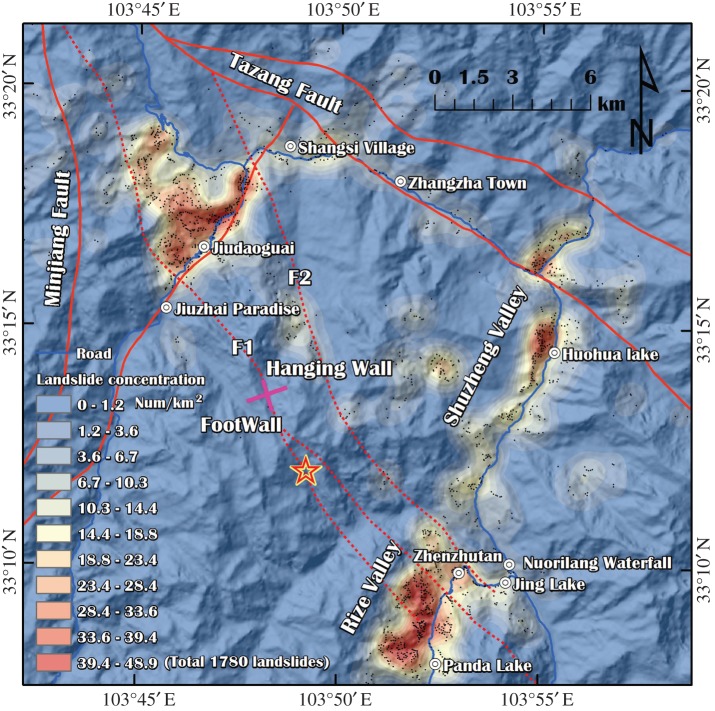
Distribution map derived from this study. The map contains points showing 1780 landslides, one broken dam and one collapsed dam.

The active faults in the northern section of the Minshan Block include the Tazang fracture and the Minjiang fault (figure 2). Based on the field investigations, the seismic fault is a branch of the Tazang fault following the NW direction and has a dip direction of approximately 87°. Seismic faults consist of two concealed faults (*F*_1_ and *F*_2_ in figure 3); the lengths of *F*_1_ and *F*_2_ are approximately 23.0 km and 19.0 km, respectively [11].

The PGA (peak ground acceleration) and peak velocity are listed as follows: EW: 129.5 cm s^−2^, NS: 185.0 cm s^−2^, UD: −124.7 cm s^−2^ and east–west (EW): 3.9 cm s^−1^, north–south (NS): 6.6 cm s^−1^, up–down (UD): 2.5 cm s^−1^ [13]. As of 14.00 on 17 August, a sum of 4799 aftershocks had been detected by the China Earthquake Monitoring Web [14]. Among these aftershocks, 31 had a magnitude of 3.0 and above, as shown in figure 2, 28 had magnitudes of 3.0–3.9 and three had magnitudes of 4.0 or greater. The strongest aftershock had a magnitude of 4.8 and occurred on 9 August. The aftershocks were concentrated in a belt zone that was 30 km long and 8 km wide following the NW direction (figure 2).

The area affected by the Jiuzhaigou earthquake was mountainous with elevations from 1600 to 4500 m, as shown in figure 2. The region affected by geohazards extended approximately 30 km in width and 35 km in length.

### 2.3. Basic description of the geohazards induced by the Jiuzhaigou earthquake

Based on field investigations and remote sensing interpretations (initial remote sensing interpretations were supported by the State Key Laboratory of Geohazard Prevention and Geoenvironment Protection [15]), the basic distributions of geohazards are shown in figure 3. Overall, 1780 landslides occurred, one dam was broken (the Huohua Lake dam) and one dam was damaged (the Nuorilang Waterfall dam) in the study region.

**Figure 4.**
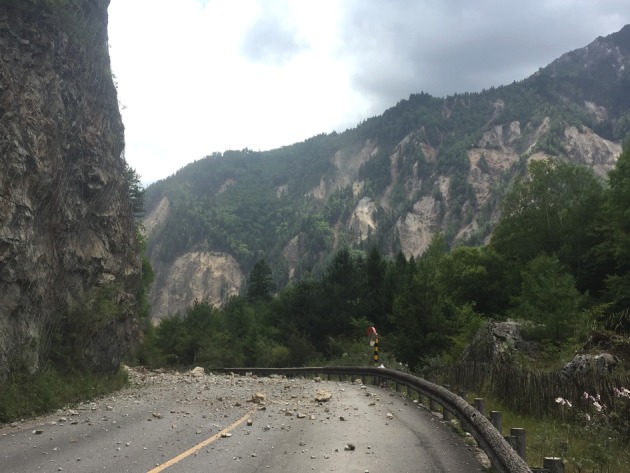
The geohazards were densely distributed on the back slope of the Rize Valley.

From figure 3, it can be observed that the main geohazard areas are concentrated in the Rize Valley, Jiuzhai Paradise and Shuzheng Valley, especially in the Panda Lake and Jiuzhai Paradise, where the geohazard concentration is highest (greater than 45 km^−2^). Other distribution laws can be summarized as follows:

(1) *Hanging wall effect.* Most geohazards are distributed in the hanging wall of the main seismic fault (*F*_1_). Relative studies indicate that the dynamic conditions in hanging walls are better than those in footwalls [16,17].
(2) *Back-slope effect.* The geohazards in the Rize Valley and Shuzheng Valley mainly occurred on the back slopes and not at the epicentre (the slope aspect is parallel to the movement direction of the seismic wave), as shown in figures 3 and 4.

The Jiuzhaigou earthquake-triggered geohazards can be classified into three broad categories:

(1) landslides,
(2) damage to the Ruorilang Waterfall dam, and
(3) breaking of the Huohua Lake dam.

## 3. General characteristics of geohazards

### 3.1. Landslides induced by the Jiuzhaigou earthquake

#### 3.1.1. Typical landslide types induced by the Jiuzhaigou earthquake

Overall, 1780 landslides were triggered by the Jiuzhaigou earthquake. Most of these landslides are small, with scales of a few cubic metres to thousands of cubic metres and few reached hundreds of thousands of cubic metres.

**Figure 5.**
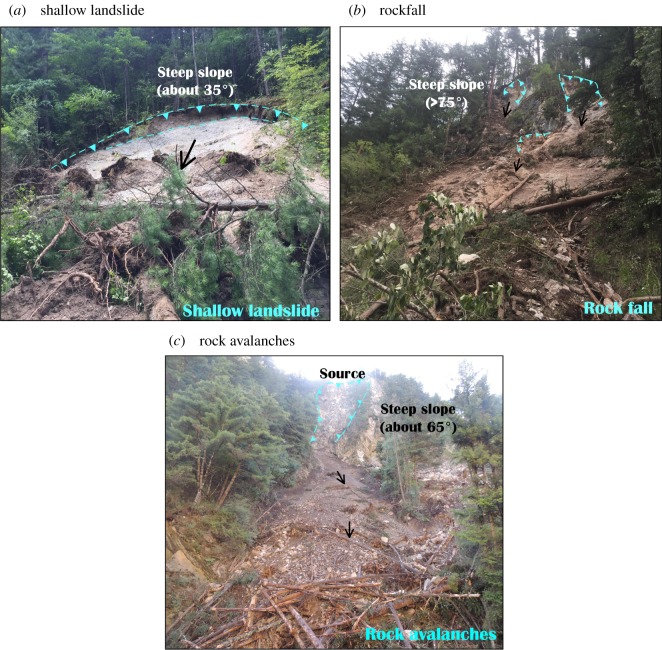
Landslides induced by the Jiuzhaigou earthquake.

Most of the landslides were shallow landslides (figure 5*a*), rockfalls from steep slopes (figure 5*b*) and rock avalanches (figure 5*c*), which typically involve the top few metres of the weathered bedrock, regolith and colluvium.

The landslides were distinguished as low-position or high-position landslides based on their position.

(1) *Low-position landslides.* This type of landslide is distributed along the bank slope of the Rize Valley and Shuzheng Valley and is associated with engineering activities at elevations between 5 and 100 m above the road. This type of landslide is the main type of rockfall and involves shallow surface sliding. The volume of the accumulation is almost tens to thousands cubic metres.
(2) *High-position landslides.* Compared with the low-position landslides, the high-position landslide is distributed on the upper part of the mountain or on the convex topography of the slope, and the elevation is between 100 and 400 m above the valley floor. This slope failure mainly includes rock avalanches and shallow landslides.

#### 3.1.2. Failure modes of landslides

**Figure 6.**
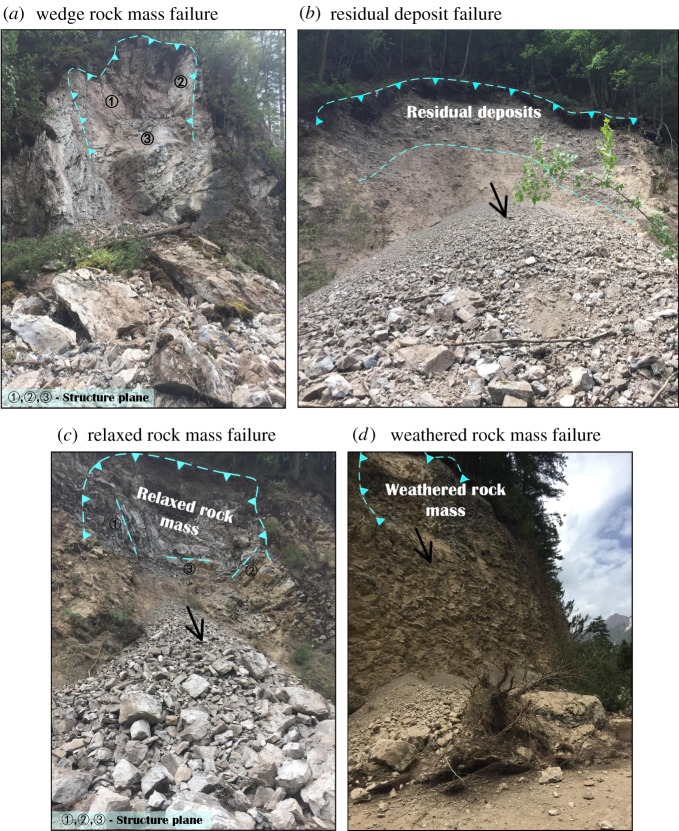
Failure modes of the landslides.

Based on field investigations and comprehensive analysis, the failure modes of landslides in earthquake areas can be summarized as follows: (i) wedge rock mass failure (figure 6*a*), (ii) residual deposit failure (figure 6*b*), (iii) relaxed rock mass failure (figure 6*c*) and (iv) weathered rock mass failure (figure 6*d*).

(1) *Wedge rock mass failure*. The structure planes cut the rock mass from different directions and turn it into a wedge rock mass. Owing to the structure planes, the wedge rock mass is in a potentially unstable state. Thus, the earthquake finally triggered the movement of these unstable rock masses (figure 6*a*).
(2) *Residual deposit failure*. In the earthquake area, the residual deposits were distributed widely, and their poor properties (low shear strength and low tensile strength) [18] result in instability. Later, engineering practices such as road construction result in residual deposits on free surfaces. Finally, the residual deposits slide off the free surfaces (figure 6*b*). Many shallow landslides follow this mode.
(3) *Relaxed rock mass failure*. In earthquake areas, many steep slopes with large amounts of relaxed rock mass are very fractured and are accompanied by developed structural planes. These rock masses collapse suddenly during earthquakes, as shown in figure 6*c*. This failure mode can trigger larger scale landslides. Most rock avalanches follow this failure mode.
(4) *Weathered rock mass failure*. Steep slopes with strong weathered rock masses appear in many road slopes. During earthquakes, these strong weathered rock masses slide down (figure 6*d*).

### 3.2. Dam damage at the Nuorilang Waterfall

**Figure 7.**
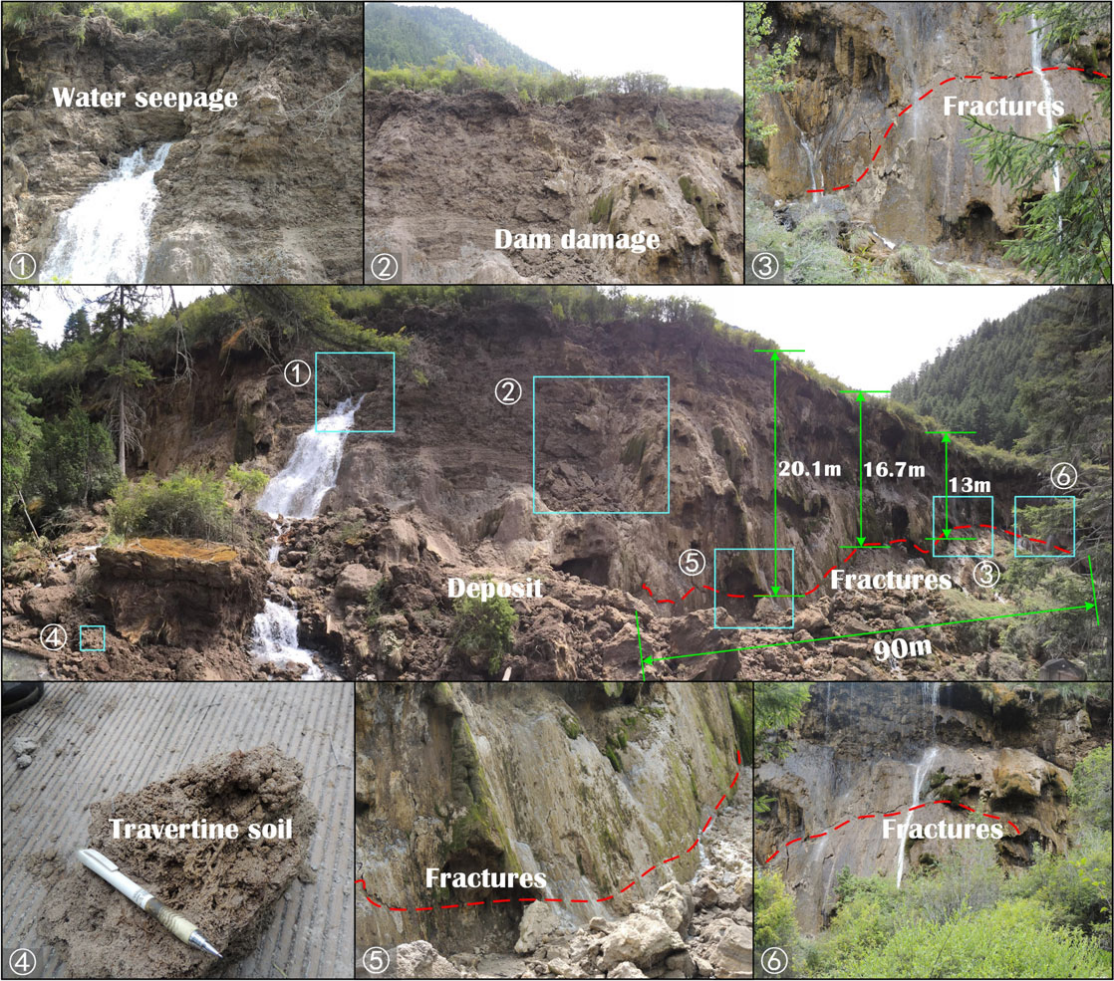
Damage of the Nuorilang Waterfall dam.

The Jiuzhaigou earthquake damaged the Nuorilang Waterfall dam, resulting in its partial collapse and in many shear cracks (figure 7). The Nuorilang Waterfall dam is a calcified dam that gradually extended by limestone deposition and is composed of travertine and algae (figure 7–4) as well as sediments with the following components: SiO (7.9%), AlO (2.2%), Fe_2_O_3_ (0.31%), FeO (0.47%), MgO (2.19%), CaO (47%), K_2_O (0.36%), Na_2_O (0.19%), Ti_2_O (0.09%), P_2_O_5_ (0.05%), MnO (0.01%), CO_2_ (36.6%) and H_2_O^+^ (2.15%) [19].

**Figure 8.**
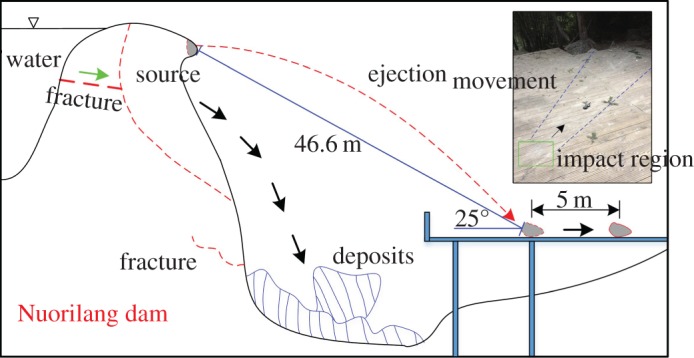
The ejection phenomenon in the Nuorilang dam.

The degree of collapse of the Nuorilang dam varied with location, especially in the western section of the dam. The most collapses occurred in the upper part of the dam, and the dimensions of the largest rock mass are approximately 4 m × 3 m × 6 m. An ‘ejection’ type of collapse also occurred in which some dam masses were ejected to the viewing platform (opposite side of dam), and the ejection length exceeded 40 m (figure 8). When masses were ejected out horizontally, the initial ejection velocity was approximately 21.1 m s^−1^. Wang *et al.* [20] indicated that the ejection phenomenon of a rock mass is an obvious sign of topographic magnification.

The earthquake also caused the dam to produce many fractures, and the lake water flowed out through these fractures (figure 7). In these fractures, an approximate horizontal shear fracture was observed with a wavelike distribution, a length of approximately 92 m and a distance to the top of the dam of 13–20 m. These fractures indicate that severe shear damage exists in the Nuorilang dam body.

### 3.3. Breaking of the Huohua Lake dam

**Figure 9.**
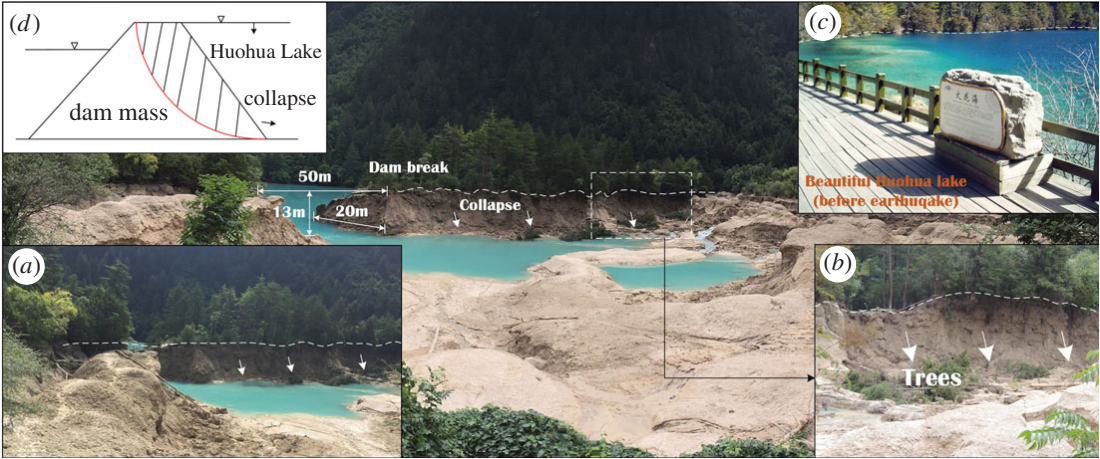
Dam break (Huohua Lake).

The Jiuzhaigou earthquake caused the Huohua Lake dam to break (figure 9). The Huohua Lake is 232 m long, 134–294 m wide and 16 m deep with a total capacity of 4.5 × 10^5^ m^3^. The Huohua Lake dam is a thin ridge dam bonded by algae and has a very loose structure.

The distance from Huohua Lake to the epicentre is only 5.3 km. During the earthquake period, the dam broke at its weakest part, which was approximately 50 m in length, 20 m in width and 12 m in height.

After the dam broke, the discharge of water increased from the normal level of 9.3 m^3^ s^−1^ to a maximum of 21.5 m^3^ s^−1^, and several hours later, the beautiful natural lake was gone. During the rapid descent of the water level in Huohua Lake, many collapses occurred along the dam (figure 9).

## 4. Landslide distributions influenced by different factors

Because landslides accounted for 99.89% of all the co-seismic geohazards, this chapter mainly focuses on the distribution of landslides in the study area.

Although co-seismic landslides are abundant around the epicentre, they are concentrated in specific zones associated with specific geology, geomorphology, topography and seismic factors [21–25]. An analysis of the causal relations between the triggering factors and geo-environmental conditions is important to comprehensively understand the landslide distribution patterns triggered by earthquakes [26].

**Figure 10.**
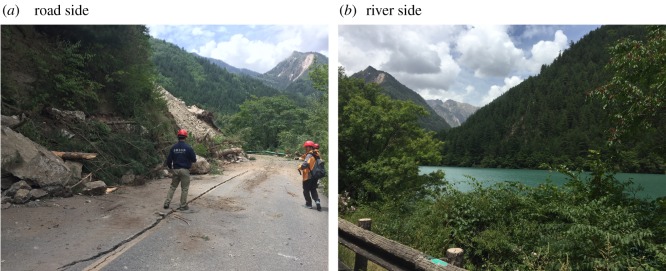
A large number of landslides triggered along the road around Jing Lake.

Although there are many influencing factors posing an influence on landslide distribution, different influencing factors may have different responses. The selection of influencing factors should be based on the actual condition in the field, for example, some studies choose the influence factor ‘distance to stream’ as one factor [27,28], while the landslides along streams triggered by Jiuzhaigou earthquake are not obvious according to field investigation; unexpectedly, a large number of landslides triggered along roads, as shown in figure 10.

In this paper, the relationships between the landslide distribution and the following influencing factors are analysed: seismic factors, slope angle, slope aspect and other factors. The landslide percentage is used to reflect the influence of landslide occurrence, which is defined as the proportion of landslides that meet the requirements for the total landslides.

### 4.1. Landslide distribution by seismic factors

Many studies have focused on the relationships between landslide concentrations and triggering seismic factors [22,24,26,29–31]. This study primarily analysed the relationships between landslide distribution and intensity and seismic faults by analysing the relationships among the landslide distribution, distance to the seismic fault and intensity.

**Figure 11.**
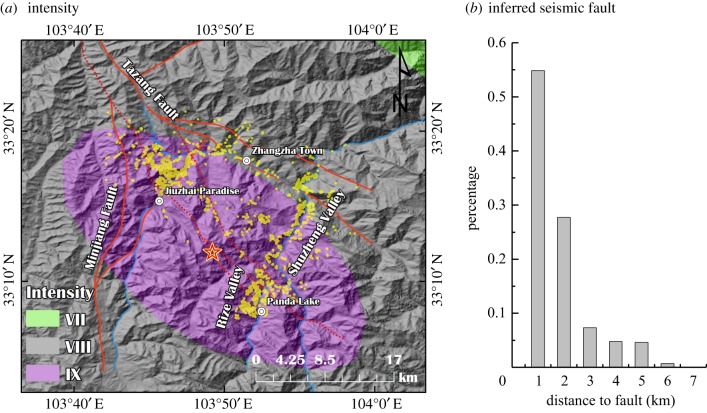
(*a*,*b*) Relationship between landslide concentration and seismic factors.

Figure 11*a* shows the variations in the distributions of landslides with different intensities. All landslides had intensities from VIII to IX, and most of the landslides had an intensity of IX.

Figure 11*b* shows the variations in the landslide distribution with distance from the inferred seismic faults. The number of landslides decreased with distance from the seismic fault, with the highest landslide concentration occurring at a distance of 2 km, which accounted for approximately 80% of the landslides. The statistical data analysis also indicated that nearly all of the landslides were located within 6 km of a seismic fault.

### 4.2. Landslide distribution by slope angle

Slope angle has a significant influence on landslide susceptibility; when all other factors are equal, steeper slopes are more susceptible to failure than flatter slopes [32]. To analyse the influences of slope angle on landslide distribution, the slope angle was calculated from the 30 m × 30 m digital elevation model (DEM). Then, a slope map was constructed with intervals of 10°.

**Figure 12.**
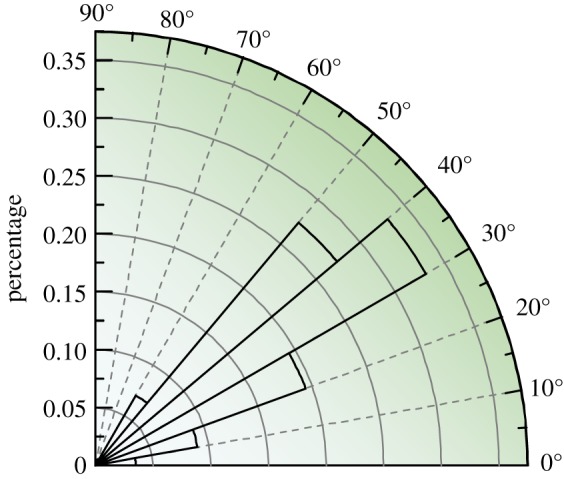
The relationship between landslide concentration and slope angle.

Figure 12 illustrates that the geohazard percentage increased as the slope angle increased was maximized at slopes of 30°−40°, and then slightly decreased at slopes of 40°−50°. The landslides in these two categories (30°−50°) account for more than 60% of the landslides. Similar results were reported by Parise & Jibson [32], Wasowski *et al.* [22] and Qi *et al.* [33] for co-seismic landslides in different parts of the world. From our field investigation, very steep slopes (more than 50°) are more prone to rockfall (figure 5*b*). However, the occurrence of the steepest inclinations (over 70°) is underrepresented. The maximum slope retrieved from the 30 m DEM did not exceed 70° [5]. As noted by Jibson *et al.* [34], this may be a common problem of digital elevation models generated from topographic maps, in which the steepest terrains are probably not accurately portrayed.

### 4.3. Landslide distribution by slope aspect

**Figure 13.**
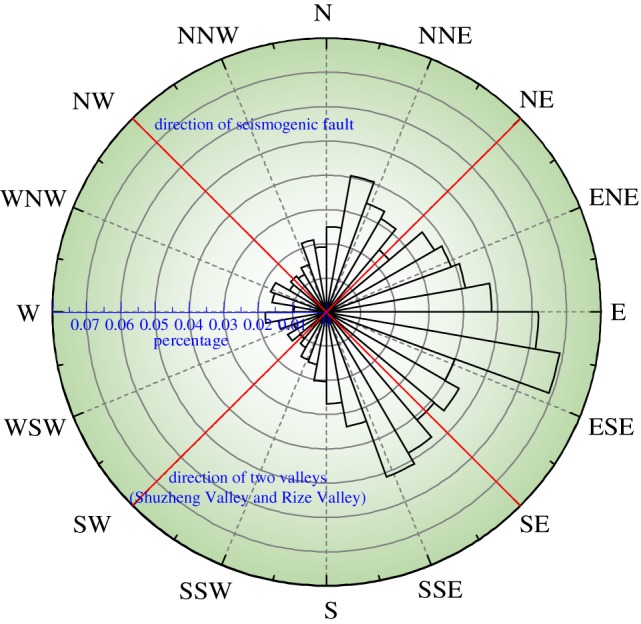
The relationship between landslide concentration and aspect.

The statistical results (figure 13) show that slope aspect plays a significant role in landslide distribution. Eastern and southeastern-facing slopes have relatively higher landslide densities than those facing other directions; these slope aspects are approximately equal to the direction of seismic fault movement and account for more than 60% of the landslides.

In the NW direction, the landslide percentage was obviously lower although this was the direction of the seismic fault. One possible explanation for this result is that two transverse valleys (Shuzheng Valley and Rize Valley) exist in the NE direction in the southern section of the epicentre. Thus, many slopes are parallel to the direction of seismic wave movement. The back-slope effect caused the landslides to be concentrated on the back slopes of the two valleys, and this obvious distribution can be found in figure 3.

### 4.4. Landslide distribution based on other influence factors

#### 4.4.1. Valley trend and distribution

Over broad regions, triggering of the landslide distribution was strongly correlated with the valley trend. For example, the Shusheng Valley follows the SE direction, which is approximately vertical to the seismic fault (*F*_1_), while the Zhangza Valley follows the NW direction, which is approximately parallel to the seismic fault. The field investigation and interpretation show that the landslide concentration of the Shuzheng Valley is obviously higher than that of the Zhangza Valley (figure 3).

**Figure 14.**
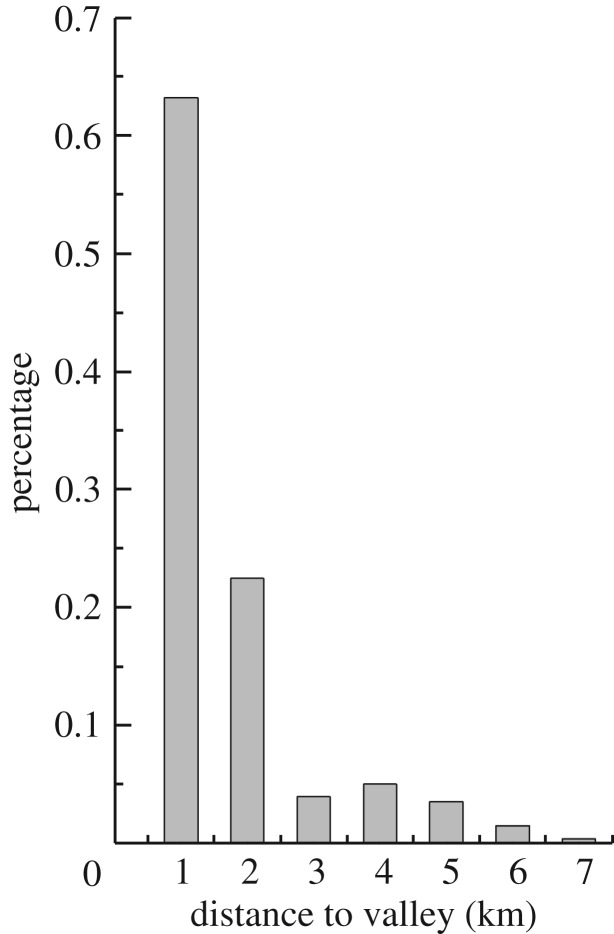
Variations in landslide concentration with distance from valley.

Figure 14 shows the landslide distribution with distance from the valley floor. As shown in Figure 14, more than 85% of the landslides occurred within 2 km of the valley floor. The valley area had the highest concentration of landslides because it provides advantageous topographic, slope and dynamic conditions.

#### 4.4.2. Road

**Figure 15.**
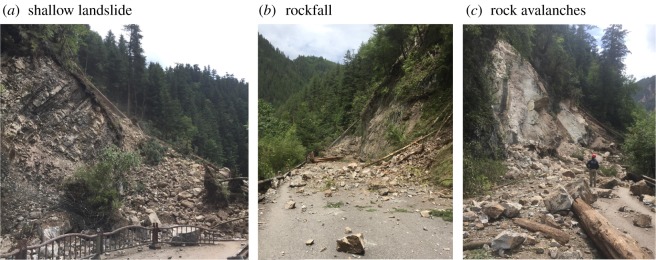
The distribution of geohazards along the roads. (*a*) Shallow landslide, (*b*) rockfall and (*c*) rock avalanches.

According to results from the emergency investigation, several landslides occurred along roads that caused considerable damage and disrupted roadway use, as shown in figure 15; for example, from Jing Lake to Zhenzhutan Waterfall, a distance of approximately 2300 m (figure 3), 28 landslides and 2 road collapses occurred. This finding might be attributed to the fact that many cut slopes are composed of weathered and heavily fractured bedrock and are thus very susceptible to seismic shaking failure. In addition, this phenomenon supports the view held by Keefer [30], Barnard *et al.* [35] and Owen *et al.* [31] that human modification of slopes by road construction is an important influential factor in the initiation of landslides in tectonically active regions.

## 5. Discussion

### 5.1. Dynamic responses of the Jiuzhaigou earthquake

**Figure 16.**
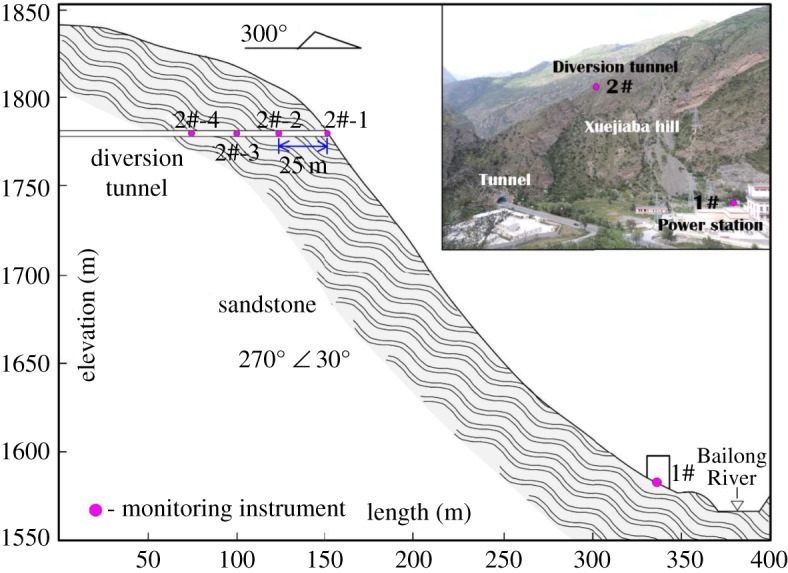
Geological profile across the Xuejiaba hill showing the positions of the monitoring stations.

The field investigations indicated that the amplification effect of seismic waves exists in many places, such as on back slopes, as discussed in §3.1. After the Jiuzhaigou earthquake occurred, our group carried out an earthquake monitoring scheme at a power station to study the dynamic response of the Jiuzhaigou earthquake, as shown in figure 16. There are five monitoring stations in the monitoring scheme: one station is located at the foot of the hill and is the reference station, and the other four stations are located 25 m apart in a diversion tunnel (figure 16). In addition, the diversion tunnel is located on the upper part of the hill with two free surfaces.

At 1.57 on 6 September 2017, the monitoring station successfully captured an aftershock with Ms 3.2 and 10 km depth (epicentre: 103.78° E, 33.26° N). Table 1 summarizes the typical dynamic response parameters of this aftershock.

**Table 1.** The dynamic response parameter characteristics at different stations.

	PGA (10^−2^ m s^−2^)			Arias *I*_a_ (10^−4^ m s^−1^)			frequency (Hz)		
station number	EW	SN	UD	EW	SN	UD	EW	SN	UD
1#	1.368	1.352	0.652	6.03	5.94	1.44	20	21	27
2#-1	2.365	2.917	1.618	17.91	27.32	8.41	4.5	4.5	6
2#-2	1.472	2.159	1.605	6.92	14.9	7.93	4	4	5.5
2#-3	1.465	1.443	1.321	6.87	6.76	5.65	2.5	3	5

From table 1, an amplification effect of the seismic wave is observed; for example, the PGA of 2#-1 is 2–3 times greater than that of the reference station (1#), and the Arias strength of 2#-1 is 3–6 times greater than that of 1#, while the frequency of 2#-1 is only approximately 22% of 1#. In addition, inside the mountain (2#-1, 2#-2 and 2#-3), the PGA and Arias decrease greatly with depth.

These results indicate that the dynamic response at the surface of the upper mountain is the most obvious. In the upper mountain, the larger PGA coupled with the longer action time (lower frequency) makes the upper mountain more unstable.

In addition, based on our previous studies, the PGA of the back slope is approximately 2–3 times greater than that of the face slope [10]. When the seismic wave meets the free surface in the forward direction, the reflection of waves allows tension cracks and failure planes to occur in the superficial rock mass [36,37].

### 5.2. Other phenomena caused by the Jiuzhaigou earthquake

As shown in figure 3, the geohazard distribution around the epicentre is less than that around the valleys. The main causes of this phenomenon are as follows:

(1) Mountains around the epicentre are mainly vigorous ridges with mainly compact limestone lithology. In addition, sparse vegetation and bare bedrock result in a low geohazard distribution in this area.
(2) The valley trend is parallel to seismic faults in a NW direction, and the earthquake is a strike-slip earthquake. Thus, the main earthquake force acts on the lateral fixed boundaries rather than the free boundaries; this dynamic condition is unfavourable for the occurrence of landslides. In addition, not much engineering activity occurred in the region, which reduced the number of potential unstable slopes.
(3) The elevation of this region is generally higher than 4000 m, and some places are covered by snow or clouds, which can cause some interference in the interpretation of the results.

In addition, the geohazards around the epicentre are mainly focused on the mountain top and prominent ridge, potentially due to topographic amplification. According to our previous studies, the PGA of prominent ridges after topographic amplification is approximately 4–11 times greater than that at the foot of the mountain [38].

The influencing factors of dam damage (dam damage of Nuorilang Waterfall and dam break of Huohua Lake) are different from landslides' partially. For dam damage, except for dynamic condition (topographic amplification), their special dam structure also plays an important role.

The causes of the Nuorilang Waterfall failure can be summarized as follows:

(1) The dam has low stability. The mechanical tests show that the dam consisted of a poor material with high porosity and low mechanical strength, which made the dam have a low stability that resulted in some rockfalls from the nearly vertical dam (with a height of more than 20 m) from time to time during the non-earthquake period.
(2) The nearly vertical dam can produce obvious topographic amplification at its upper part, and because the dam is located in the influence area of the back-slope effect, the amplification effect is strengthened further. The excess pore water pressure can also be produced in the dam mass in the dynamic process. In addition, the free surfaces provide enough space for deformation and final collapse.

Coupled with these influencing factors, the Nuorilang Waterfall dam collapses from the top.

Causes of Huohua Lake dam failure can be summarized as follows:

(1) Excess pore water pressure makes the stability of dam decrease greatly. Because the dam is made up of loose soil, the shaking caused by earthquake makes the pore water pressure increase rapidly and this decreases the shear strength of dam soil.
(2) Loose soil also has topographic amplification. One time we put 1# station on the nearby accumulations and we obtained a set of seismic data, as listed in table 2. From table 2, the PGA and Arias strength of 1# station are obviously larger than the upper part of the mountain, this indicates that the loose accumulations also have topographic amplification. In addition, the earthquake caused lake water to produce huge kinetic energy difference due to elevation difference (approximately 10 m) between two sides of the dam (figure 9*d*).

Coupled with the above points, these influences of excess pore water pressure, topographic amplification and kinetic energy difference etc., the dam of Huohua Lake collapsed finally.

**Table 2.** The dynamic response parameter characteristics of different stations (T-2). Note: these data were obtained from an Ms 4.5 earthquake (103.79° E, 33.21° N) that occurred at 5.31, 7 November 2017 with a focal depth of 19 km.

	PGA (10^−2^ m s^−2^)			Arias *I*_a_ (10^−4^ m s^−1^)			frequency (Hz)		
station number	EW	SN	UD	EW	SN	UD	EW	SN	UD
1#	22.0	17.2	16.2	30	20	20	10.63	8.78	8.57
2#-1	6.0	10.8	8.6	5	9	7	4.71	10.93	9.97
2#-2	4.5	13.3	5.5	2	7	3	4.55	2.82	6.02
2#-3	4.7	6.2	5.5	2	4	3	3.02	2.89	6.16

### 5.3 The typical features of the landslides of the Jiuzhaigou earthquake compared with those of recent earthquakes

In the last 10 years, the 2008 Wenchuan Ms 8.0 earthquake and the 2013 Lushan Ms 7.0 earthquake occurred along the Longmenshan fault zone at the eastern edge of the Qinghai-Tibet Plateau and caused several landslides. However, the features of landslides induced by the Jiuzhaigou earthquake are different from those by these two earthquakes.

(1) Compared with the 2008 Wenchuan Ms 8.0 earthquake

The PGA recorded in the valley bottom, the meizoseismal area of a strong earthquake in the Wenchuan earthquake, is over 0.5 g (table 3). If the source area is at the high position of the mountain, the PGA of the source area after the topographic amplification may reach 1–2 g. Under this condition, the Wenchuan earthquake generated many extremely large and deep-seated landslides and rock avalanches with volumes of tens of millions of cubic metres [3].

**Table 3.** The PGA of different earthquakes [5,13,39].

	EW	NS	UD
earthquake	PGA (cm s^−2^)	PGA (cm s^−2^)	PGA (cm s^−2^)
Wenchuan Ms 8.0	957.7	652.9	948.1
Lushan Ms 7.0	380.0	310.0	230.0
Jiuzhaigou Ms 7.0	129.5	185.0	124.7

In the Jiuzhaigou area, most of the landslides were relatively small, and no landslides had a volume exceeding 1 million m^3^. The reason for the lack of large, deep landslides triggered by the Jiuzhaigou earthquake probably relates to the differences in duration and frequency between the two earthquakes. The Wenchuan earthquake had a much larger magnitude, which resulted in a longer duration and longer period of shaking than the Jiuzhaigou earthquake.

(2) Compared with the 2013 Lushan Ms 7.0 earthquake

Although the 2013 Lushan earthquake and the 2017 Jiuzhaigou earthquake are both Ms 7.0 earthquakes, the Lushan earthquake is a thrusting earthquake, and the Jiuzhaigou earthquake is a strike-slip earthquake. The Lushan earthquake is more intense than the Jiuzhaigou earthquake (table 3). Approximately 3800 landslides in an area of 13 323 km^2^ were induced by the Lushan earthquake [32], and approximately 1780 landslides in an area of 1050 km^2^ were induced by the Jiuzhaigou earthquake. Although the earthquake area and landslides of the Lushan earthquake are both much larger than those of the Jiuzhaigou earthquake, the landslide concentration of the Jiuzhaigou earthquake is higher than that of the Lushan earthquake. In addition, the hanging wall effect and back-slope effect of the Jiuzhaigou earthquake are also more obvious than those of the Lushan earthquake.

## 6. Conclusion

The 2017 Jiuzhaigou earthquake with a magnitude of Ms 7.0 occurred in the eastern segment of the Tazang fault zone in Sichuan, China. The epicentre is located in the Jiuzhaigou scenic spot.

The seismic fault is a branch of the Tazang fault following the NW direction and is made up of two concealed faults with lengths of approximately 23.0 and 19.0 km. The earthquake triggered 1782 geohazards, including 1780 landslides, one damaged dam (Nuorilang Waterfall) and one broken dam (Huohua Lake). The main types of landslides included shallow landslides, rockfalls and rock avalanches, whose scales were mainly from a few cubic metres to thousands of cubic metres, and a few landslides reached a scale of hundreds of thousands of cubic metres. Most of the landslides occurred along valleys and were concentrated on the hanging wall of the seismic faults. The landslide concentration was positively correlated with the slope angle and the distances from the seismic fault and valleys.

In addition, the Jiuzhaigou scenic spot is a world natural heritage site and is famous for its beautiful scenery. Although this earthquake produced some damage to the Jiuzhaigou scenic spot, such as Huohua Lake and the Nuorilang waterfalls, which may take a long time to recover, most other scenic spots remain intact. In addition, after this earthquake, many mountains may produce dangerous rock masses; thus, detailed geological investigation is necessary in the future.

## Supplementary Material

Remote senic interpretation

## Data accessibility

Data concerning results of remote scenic interpretation are available in the electronic supplementary material. The seismic fault can be found in Li *et al.* [11]. The GPS velocities were downloaded from [9]. The remaining data can be found within the article.
